# A Bivalent Trans-Amplifying RNA Vaccine Candidate Induces Potent Chikungunya and Ross River Virus Specific Immune Responses

**DOI:** 10.3390/vaccines10091374

**Published:** 2022-08-23

**Authors:** Christin Schmidt, Florian D. Hastert, Julia Gerbeth, Tim Beissert, Ugur Sahin, Mario Perkovic, Barbara S. Schnierle

**Affiliations:** 1 Department of Virology, Paul-Ehrlich-Institut, Paul-Ehrlich Strasse 51-59, 63225 Langen, Germany; 2 TRON (Translational Oncology at the University Medical Center), Johannes Gutenberg University Mainz, Freiligrathstraße 12, 55131 Mainz, Germany; 3 Research Center for Immunotherapy (FZI), University Medical Center, Johannes Gutenberg University, Langenbeckstr. 1, 55131 Mainz, Germany

**Keywords:** chikungunya virus, Ross River virus, alphavirus, RNA vaccine, replicon, taRNA

## Abstract

Alphaviruses such as the human pathogenic chikungunya virus (CHIKV) and Ross River virus (RRV) can cause explosive outbreaks raising public health concerns. However, no vaccine or specific antiviral treatment is yet available. We recently established a CHIKV vaccine candidate based on trans-amplifying RNA (taRNA). This novel system consists of a replicase-encoding mRNA and a trans-replicon (TR) RNA encoding the antigen. The TR-RNA is amplified by the replicase in situ. We were interested in determining whether multiple TR-RNAs can be amplified in parallel and if, thus, a multivalent vaccine candidate can be generated. In vitro, we observed an efficient amplification of two TR-RNAs, encoding for the CHIKV and the RRV envelope proteins, by the replicase, which resulted in a high antigen expression. Vaccination of BALB/c mice with the two TR-RNAs induced CHIKV- and RRV-specific humoral and cellular immune responses. However, antibody titers and neutralization capacity were higher after immunization with a single TR-RNA. In contrast, alphavirus-specific T cell responses were equally potent after the bivalent vaccination. These data show the proof-of-principle that the taRNA system can be used to generate multivalent vaccines; however, further optimizations will be needed for clinical application.

## 1. Introduction

Alphaviruses are part of the Togaviridae family. The different members are globally distributed and are transmitted to various vertebrate hosts by mosquito vectors. Traditionally, alphaviruses are classified into “Old World” and “New World” viruses. In general, infection with New World alphaviruses such as Western, Eastern, and Venezuelan equine encephalitis viruses (WEEV, EEEV and VEEV) can cause encephalitic diseases, while infection with Old World alphaviruses causes arthritogenic diseases.

Alphaviruses are single-stranded positive-sensed RNA viruses. Their genome consists of two open reading frames, which encode for either the non-structural proteins (nsPs) or the structural proteins. After infection of a host cell, the first open reading frame of the genomic RNA is directly translated and the four nsPs (nsP1–nsP4) assemble into the alphavirus replicase. The replicase synthesizes a full-length negative-sensed RNA, from which the genomic and subgenomic RNA are transcribed. The structural proteins (capsid and the envelope proteins E3-E2-6K-E1) are translated from the subgenomic RNA and their expression is under control of the subgenomic promotor (SGP) [1]. The envelope proteins E1 and E2 are embedded in the viral membrane [2]. The E2 protein is involved in receptor binding and is the target of neutralizing antibodies [3].

Among the different members of the Old World alphaviruses are the human pathogenic chikungunya virus (CHIKV), Ross River virus (RRV), and Mayaro virus (MAYV). CHIKV rapidly spread worldwide after the vector extension from *Aedes aegypti* to *Aedes albopictus*, and it has now been identified in over 100 countries and has caused multiple epidemics [4,5,6]. RRV has caused epidemics in Australia, Papua New Guinea, and the South Pacific islands and is the most common arboviral disease in Australia [7]. A further spread of CHIKV and RRV due to globalization and climate change is both possible and very likely [8]. Acute CHIKV and RRV infections are characterized by high fever, rash, myalgia, and polyarthralgia [9]. Symptoms usually resolve after two weeks; however, disease can become persistent in CHIKV and RRV patients with severe joint pain and arthritis lasting for months to years [10,11].

Different vaccine candidates against CHIKV and RRV have been studied in vitro and in vivo, and although some are promising, none has yet been licensed [12,13,14]. We recently generated a new CHIKV vaccine candidate based on trans-amplifying RNA (taRNA) [15]. This vaccine candidate utilizes two RNAs: the first is a synthetic mRNA encoding for the CHIKV replicase and the second is the trans-replicon (TR) RNA, which encodes for the CHIKV envelope proteins. The TR-RNA contains the CHIKV 5′ and 3′ conserved sequence elements (CSEs) that allow the replicase to specifically amplify the TR-RNA [1,16]. Thus, a potent immune response can be induced with a low amount of antigen-encoding RNA. The concept of taRNA has been studied before in vitro, for example for the functional analysis of the CHIKV replicase proteins [17,18]. In a similar approach, a VEEV splitzicon system demonstrated that the 5′ CSE, the SGP and the untranslated regions are required for specific RNA amplification by the replicase [19]. Moreover, in vitro experiments showed that the alphavirus replicase preferentially amplifies shorter RNAs [15,16,20]. As a first taRNA vaccine, the combination of a Semliki Forest virus replicase RNA with a TR-RNA encoding the influenza hemagglutinin protein could induce protective immunity [21]. Here, the usage of a non-replicating replicase RNA in comparison to a self-amplifying (sa) replicase RNA resulted in a more potent immune response [21]. Moreover, in comparison to cis-encoded saRNA, where both the replicase and the antigen are encoded on the same RNA, less antigenic RNA was required for similar immune responses [21]. We observed before that even with a two-RNA system, where the replicase is encoded by an RNA containing viral cis-acting sequences and the envelope proteins by a separate TR-RNA, recombination of the RNAs to fully replication-competent virus occurred [15]. Therefore, we focused on the expression of two antigens by TR-RNAs to generate safe, bivalent vaccines.

Recently, a phase-I clinical study could show the induction of immune responses against the New World alphaviruses WEEV, EEEV, and VEEV using a trivalent virus-like particle vaccine [22]. However, in some studies, vaccination with multiple alphaviruses either sequentially or on the same day has been associated with immune interference mechanisms that resulted in lower neutralizing antibody response rates [23,24]. We were interested in determining whether the taRNA system could be used to amplify multiple antigen-encoding TR-RNAs by the replicase to generate a vaccine candidate with an extended applicability.

Here, we adapted the CHIKV taRNA vaccine candidate for a combined vaccine against the human pathogenic CHIKV and RRV. We could demonstrate that when the CHIKV and RRV envelope proteins were encoded on two separate TR-RNAs, both TR-RNAs were amplified by the CHIKV replicase, resulting in a high antigen expression in vitro. Moreover, vaccination of BALB/c mice with the taRNAs induced humoral and cellular immune responses in vivo. Similar T cell responses could be observed against CHIKV and RRV. In contrast, the total antibody titers and neutralization capacity were reduced after the bivalent vaccination in comparison to a single vaccination.

## 2. Materials and Methods

### 2.1. Cell Culture

All cells were cultured in medium supplemented with 10% fetal bovine serum (PAA, Pasching, Austria), 1% L-glutamine (200 mM; Lonza, Verviers, Belgium), and 1% penicillin/streptavidin (Fisher Scientific, Schwerte, Germany) in a humidified atmosphere with 5% CO_2_ at 37 °C. Cell lines were originally obtained from ATCC (American Type Culture Collection. HEK 293T (ATCC CRL-11268) and Vero E6 cells (ATCC CRL-1586) were grown in Dulbecco’s modified Eagle’s medium (DMEM) (Lonza, Verviers, Belgium), and BHK 21 (ATCC CCL-10) and isolated mouse splenocytes in Roswell Park Memorial Institute (RPMI) medium (Biowest, Nuaille, France).

### 2.2. Mouse Experiments

Female, 6–8 week-old BALB/c_Rj mice (Janvier, Saint-Berthevin Cedex, France) were immunized by intradermal injection on day 0 and day 28. The respective RNAs were diluted in 20 µL RNase-free PBS. Blood was collected on day 0 and day 28 by retro-orbital bleeding and final blood was collected on day 56. Splenocytes were harvested on day 56.

### 2.3. Ethics Statement

Animal experiments were performed after approval of the Darmstadt regional council, Germany (animal license no. F107/1062; Regierungspräsidium Darmstadt, Germany) in compliance with legal requirements (German protection of animals act and experimental animal regulation).

### 2.4. RNA Vectors and In Vitro Transcription

CHIKV sequences are based on the isolate LR2006 OPY1 (isolated from a French patient returning from Reunion Island during the 2006 outbreak [1,25]. The generation of the non-replicating-replicase-RNA, the TR-RNA encoding the CHIKV envelope proteins, and the TR-luc-RNA has been described previously [15]. For the TR-RNA encoding the RRV envelope proteins, the RRV E3-E1 was amplified by fusion PCR from a cDNA encoding for RRV (T48 RRV strain), which was a kind gift from David A. Sanders, Purdue University, West Lafayette, USA [26]. For the first amplicon primer f278-1 and r278-1 were used and for the second f278-2 and r278-2 (f278-1: GCAAGTATCTAAACACTAATCAGCTACACTATGCCACATGTCTGCCGCGCTGA-TG; r278-1: CCTAAACTCCTATAGGAGAACAGCGTCGG; f278-2: CCGACGCTGTTCTCCTATAGGAGTTTAGG; r278-2: GACACATATACCTTCATACTTAATTGTCAAGCGGCCGCCTCGAGGTTACCGACGCATTGTTATGCAGGTTACC). The amplicons were fused by PCR with primers f278-1 and r278-2 and used to replace the CHIKV E3-E1 on the TR template via *SpeI* and *XhoI* to generate the plasmid encoding the TR-R-RNA. The sequence of TR-R between the T7 promotor (TAATACGACTCACTATAG) and polyA is given in Supplementary Figure S1. The TR-RNA encoding the CHIKV E3-E1 proteins was previously named TR-S; for simplification, it is now named TR-C. RNA in vitro transcriptions were performed as described before [27]. Prior to in vitro transcription template plasmids were linearized using the type IIS restriction enzyme SapI which generates an unmasked poly (A) tail. Synthesis and purification of RNA were previously described [8].

### 2.5. Virus

The viruses used in this study were: CHIKV of the La Réunion strain (GenBank: DQ443544.2; kind gift from Matthias Niedrig, Robert-Koch-Institut, Berlin, Germany [28]), RRV T48 strain (kind gift from Richard Kuhn [29]), and MAYV strain TC652 (Public Health England Culture Collections; ECACC No. 0906281v). The viruses were expanded in BHK 21 cells and concentrated 100-fold by ultracentrifugation (28,000 rpm, 2 h, 4 °C). The titers in pfu/mL were calculated from plaque titration on Vero E6 cells.

### 2.6. RNA Transfection

HEK 293T cells were transfected 24 h after seeding with the respective RNAs using Lipofectamine MessengerMAX according to the manufacturer’s protocol (Thermo Fisher Scientific, Darmstadt, Germany). To evaluate RNA amplification and cellular protein expression, 0.8 × 10^6^ HEK 293T cells were transfected with 2.5 µg total RNA in 6-well plates. For the analysis of cell supernatants, 6 × 10^6^ HEK 293T cells were transfected with 10 µg total RNA in 10 cm dishes. The ratio of replicase-RNA to TR-RNA was 4:1.

### 2.7. RT-qPCR

TR-RNA amplification was quantified by RT-qPCR, as described previously [15]. Briefly, HEK 293T cells were transfected with the indicated RNAs or infected with CHIKV or RRV (MOI 3). RNA was extracted after 6 h, 16 h, and 24 h. RT-qPCR was performed with specific primers and fam-labeled probes (CHIKV E2 RNA: CHIKV E2 fw 5′-CAT GCT ACT GTA TCC TGA CCA C-3′, CHIKV E2 rev 5′-ATG GGC TGT ACC GTT TGT AG-3′, CHIKV E2 probe 5′-TGC TAA CCG TGC CGA CTG AAG G-3′; RRV E2 RNA: RRV E2 fw 5′-ACA CTT CAT CGT CGC ACA TTG TCC-3′, RRV E2 rev 5′-CTA ACC ACG AAC TTC TCT CTA CCC ACC-3′, RRV E2 probe 5′-CGA CTA CCT CAA GGT TTC GTT CGA GGA CGC A-3′), and normalized to GAPDH (PrimePCR Probe Assay: GAPDH, Human; Bio-Rad, Munich, Germany).

### 2.8. Western Blot Analysis

Protein expression was evaluated from cell lysates or concentrated supernatants (ultracentrifugation at 25,000 rpm for 1 h) by Western blot analysis. Protein concentrations were determined with the Pierce BCA Protein-Assay Kit (Thermo Fisher Scientific, Schwerte, Germany) and 20 µg protein per sample were separated by SDS-PAGE and transferred onto polyvinylidene difluoride (PVDF) membranes. Primary antibodies were directed against CHIKV E2 (Eurogentec, Cologne, Germany; custom made), RRV E2 (ATCC VR-1246AF), and β-actin (Sigma, Munich, Germany; no. A5441). After incubation with appropriate secondary horseradish peroxidase (HRP)-coupled antibodies, proteins were detected with the ECL detection system (Amersham, Freiburg, Germany) and the Fusion FX7 imaging system (Vilber, Eberhardzell, Germany).

### 2.9. ELISA

Virus-binding antibodies were detected by ELISA as described previously [15]. Briefly, ELISA Nunc-Immuno 96-well MaxiSorp ELISA plates (Thermo Fisher Scientific, Darmstadt, Germany) were coated overnight with 10^6^ pfu virus (CHIKV, RRV or MAYV) per well. For IgG endpoint titer determination, serially diluted serum samples (1:100, 1:250, 1:1000, and 1:5000) were added for 1 h at 37 °C. For isotype determination, serum samples were diluted 1:100. Respective anti-mouse secondary HRP-coupled antibodies were added for 1 h at room temperature prior to detection with TMB substrate (Mabtech, Stockholm, Sweden).

### 2.10. Neutralization Assay with Pseudotyped Lentiviral Vector Particles

Neutralizing antibodies were analyzed as described previously [30]. In brief, 6000 HEK 293T cells were seeded per well in white CELLSTAR 384-well microtiter plates (Greiner Bio-One, Frickenhausen, Germany). CHIKV- or RRV-pseudotyped lentiviral vector particles were mixed with heat-inactivated mouse serum in a final dilution range of 1:60 to 1:14,580. Luminescent signals were detected after 20 h of incubation.

### 2.11. ELISpot

Murine IFN-γ ELISpot assays were performed to assess virus-reactive T cells after vaccination. The mouse IFN-γ ELISpot Kit (BD Biosciences, Franklin Lakes, NJ, USA) and HRP streptavidin (BD Biosciences) were used following the manufacturer’s instructions. On multiscreen immunoprecipitation (IP) ELISpot PVDF 96-well plates (Merck Millipore, Darmstadt, Germany), 10^6^ splenocytes per well were stimulated with CHIKV, RRV, or MAYV at an MOI of 5 for 22 h at 37 °C. For detection, TMB substrate for ELISpot (Mabtech, Stockholm, Sweden) was used and spots were counted with an Eli.Scan ELISpot scanner (AE.L.VIS, Hamburg, Germany).

### 2.12. Statistical Analysis

Mean values and standard deviations were calculated with Excel. ELISA IgG endpoint titers were determined from serial serum dilutions by a four-parameter sigmoidal regression curve with GraphPad Prism 7.04 software (La Jolla, CA, USA). Cutoff values to interpolate the endpoint titers were based on the optical density (OD) values from naïve control sera (average at lowest dilution + four standard deviations). Samples which were below the cutoff were set to 10 for plotting and statistical analysis. The neutralization capacity of serum samples is given as the reciprocal half-maximal inhibitory concentration (IC_50_), which represents the dilution factor required to obtain 50% inhibition of lentiviral vector particle transduction. IC_50_ values were calculated as a nonlinear regression (log(inhibitor) versus response (three parameters, constrain equal to 0)) using the GraphPad Prism 7.04 software. To analyze data, one-way or two-way analysis of variance (ANOVA) with appropriate post-test was performed with GraphPad Prism 7.04 software.

## 3. Results

### 3.1. The CHIKV Replicase Efficiently Amplifies TR-CHIKV- and TR-RRV-RNA

**Figure 1:**
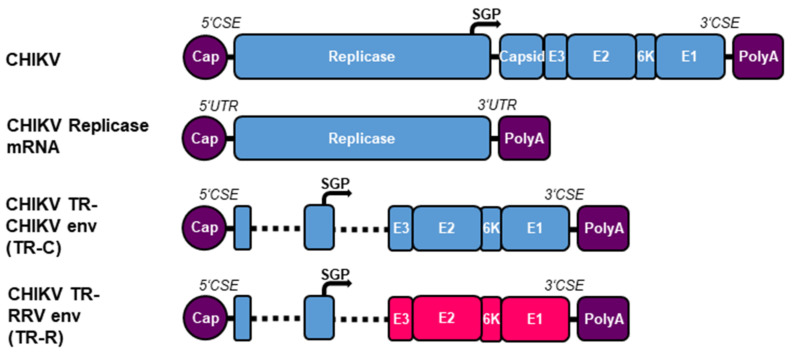
Schematic representation of the RNA constructs. The CHIKV replicase is encoded on a non-replicating mRNA with optimized 5′ UTR and 3′ UTR [31]. The CHIKV and RRV envelope proteins are encoded on TR-RNAs under the control of the CHIKV SGP (TR-C and TR-R). Both TR-RNAs contain the CHIKV CSEs, the first 231 nt of nsP1, and the last 1818 nt of nsP4. CHIKV sequences are indicated in blue and RRV in red.

A taRNA vaccine candidate consisting of a non-replicating RNA encoding the CHIKV replicase and a TR-RNA encoding the CHIKV envelope proteins (TR-C) was able to induce a potent and protective immune response in mice [15]. To extend this concept to RRV, the CHIKV envelope proteins in the CHIKV TR template were replaced by the RRV envelope proteins (TR-R) (Figure 1). Both TRs contain the CHIKV CSEs ensuring specific amplification by the CHIKV replicase [1].

**Figure 2:**
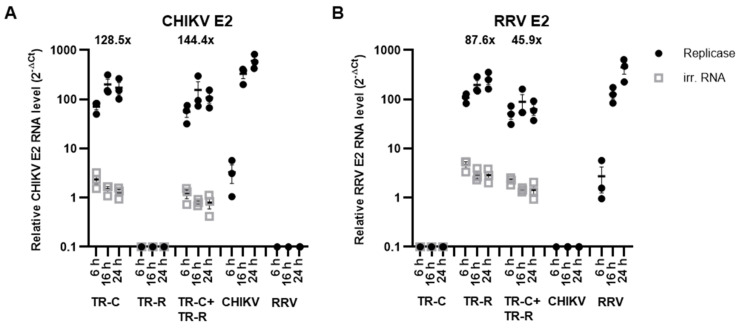
TR-RNA amplification by the CHIKV replicase. HEK 293T cells were transfected with 2 μg of the replicase RNA or TR-luc-RNA as irrelevant RNA together with 0.5 μg of the indicated TR-RNAs. The total amount of TR-RNA was kept constant between the single and double transfections. For comparison, cells were infected with CHIKV or RRV (MOI 3). RNA was harvested after 6 h, 16 h, and 24 h, and (**A**): CHIKV E2 and (**B**): RRV E2 RNA levels were measured by RT-qPCR. Numbers indicate the fold change in TR-RNA amount 24 h after co-transfection with the replicase. Ct values, which were below the cutoff, were set to 0.1 for plotting. Data are mean values ± SEM of three independent experiments.

TR-RNA amplification rates by an alphavirus replicase depends on RNA length, and a shorter RNA is preferentially amplified [15,16,20]. Here, both TR-RNAs are comparable in length and should be similarly amplified. To analyze TR-RNA amplification, HEK 293T cells were transfected with the replicase-RNA or an irrelevant RNA together with the TR-RNAs, individually or in combination. The total transfected TR-RNA amount was kept constant, resulting in the usage of half the respective TR-RNA amount in the combined TR-RNA transfection compared to the single transfections. The CHIKV E2 and RRV E2 RNA levels were quantified and normalized to GAPDH after 6 h, 16 h, and 24 h by RT-qPCR. For comparison, cells were infected with CHIKV or RRV (MOI 3). As expected, the replicase amplified both TR-RNAs (Figure 2). Without co-transfection of the replicase-RNA, CHIKV and RRV E2 TR-RNA levels declined over time. Both TR-RNA levels potently increased by 6 h after transfection with the replicase and increased further by 16 h before reaching a plateau. The CHIKV E2 TR-RNA was amplified to similar levels after transfection of the TR-C-RNA alone or in combination with TR-R-RNA (fold change 126.5× or 144.4×, respectively) (Figure 2A). Regarding the RRV E2 TR-RNA, the fold change in TR-RNA levels due to the replicase in the TR-RNA combination was slightly reduced, increasing 87.6-fold as a single RNA and 45.9-fold in the combination (Figure 2B). The CHIKV E2 and RRV E2 RNA levels observed after 16 h in virus-infected and RNA-transfected cells were similar or only slightly decreased.

### 3.2. High Antigen Expression from TR-CHIKV- and TR-RRV-RNA

**Figure 3:**
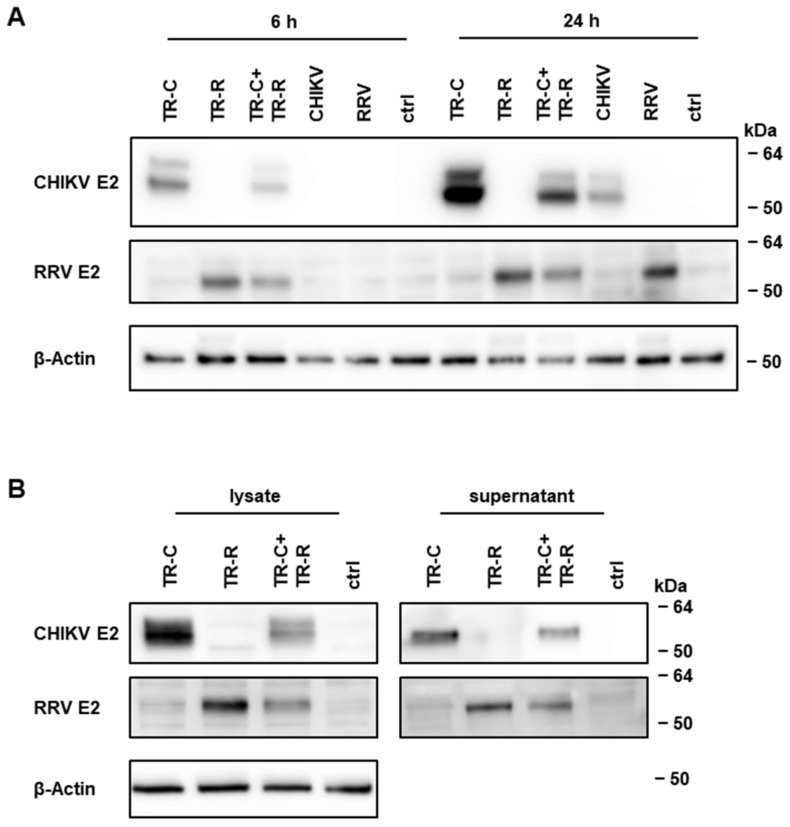
Antigen expression from TR-RNAs. (**A**): CHIKV E2 and RRV E2 protein expression in cellular lysates 6 h and 24 h after transfection of 2 μg of replicase-RNA together with 0.5 μg of the indicated TR-RNAs. As control, cells were infected with CHIKV or RRV (MOI 3) or left untreated (ctrl). (**B**): Protein expression 48 h after transfection of 8 μg of the replicase-RNA with 2 μg of the indicated TR-RNAs in cellular lysates or concentrated supernatants. The depicted Western blots are representative of three independent experiments. Uncropped blots and densitometry readings are given in Supplementary Figures S2–S6.

To evaluate if the antigens are also efficiently expressed from both TR-RNAs, expression of the CHIKV and RRV E2 proteins 6 h and 24 h after transfection/infection was studied by Western blot analysis. Expression of both proteins could already be detected 6 h after transfection and, in line with RNA amplification, this expression increased by 24 h (Figure 3A). Both E2 proteins were more strongly expressed after transfection of the respective TR-RNA alone compared to the combination of both TR-RNAs. Nevertheless, a high antigen expression could be detected in cells that had been transfected with the replicase-, TR-C-, and TR-R-RNA, and this expression even reached levels similar to those of CHIKV- or RRV-infected cells. Moreover, CHIKV and RRV E2 proteins were released into the cell supernatant (Figure 3B).

In summary, the combination of the TR-C- and TR-R-RNAs in a bivalent taRNA system demonstrated efficient RNA amplification by the replicase, which resulted in a high antigen expression in vitro. Since protein expression of the TR-RNA combination was only slightly reduced in comparison to transfection of a single TR-RNA, it encouraged us to study the bivalent alphavirus vaccine candidate further in vivo.

### 3.3. Antibody Responses after taRNA Vaccination

**Figure 4:**
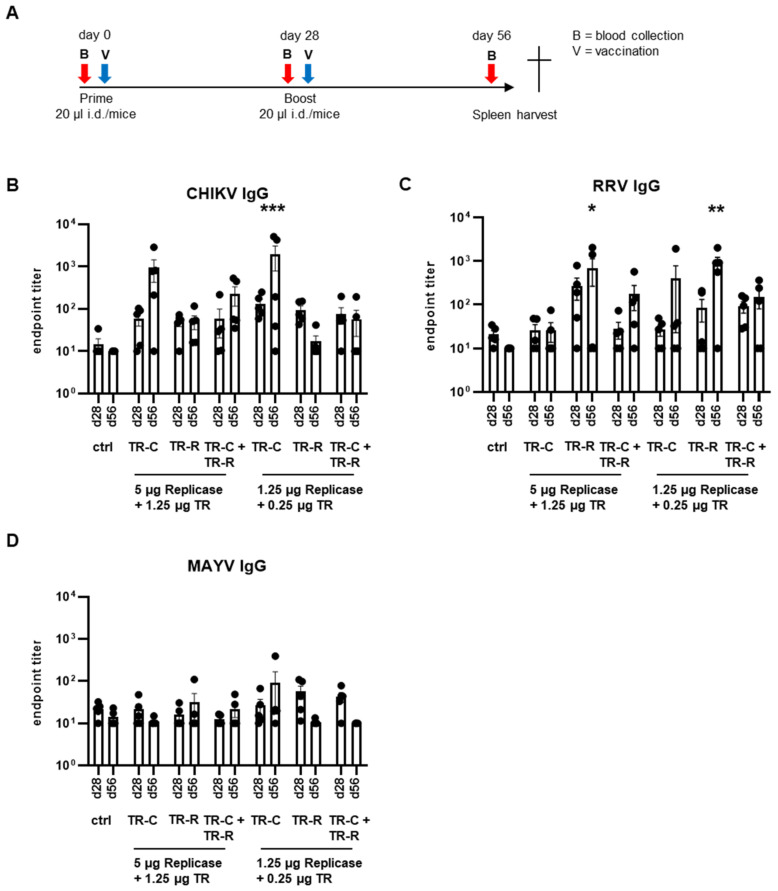
Humoral immune responses after taRNA vaccination. (**A**): Graphic illustration of the vaccination scheme. For vaccination, mice received the respective RNAs on day 0 and day 28 intradermally. As negative control, mice received PBS. For the analysis of humoral immune responses, blood was collected on day 28 and day 56. (**B**): CHIKV-binding, (**C**): RRV-binding, and (**D**): MAYV-binding IgG antibodies in mouse sera. Endpoint titers were calculated and were set to 10 for samples that were below the cutoff. The RNA doses and combinations are indicated. The mean values ± SEM of each group (*n* = 5) are depicted. For statistical analysis, a two-way ANOVA with Dunnett’s multiple comparison post-test was performed (* *p* < 0.05; ** *p* < 0.01; *** *p* < 0.001).

To study the potential of the bivalent taRNA system to induce immune responses against both encoded antigens in vivo, BALB/c were immunized intradermally with a homologous prime-boost vaccination on day 0 and day 28 (Figure 4A). As vaccine dose, mice received 5 µg or 1.25 µg replicase-RNA together with 1.25 µg or 0.25 µg total TR-RNA, diluted in RNase-free PBS. The TR-RNA combinations contained half the dose of the individual TR-RNAs. As negative control, mice received only PBS. For the analysis of humoral immune responses, blood was collected prior to the boost on day 28 and finally on day 56.

To study potential immune interferences, the immune responses of mice that had received only the TR-C-RNA, only the TR-R-RNA, or both TR-RNAs were compared. Firstly, the total CHIKV- and RRV-binding antibodies in mouse sera were measured by ELISA. As expected, CHIKV-specific IgG was detected in mice vaccinated with the replicase- and TR-C-RNA, and the antibody titers were boosted by the second vaccination (Figure 4B). Additionally, mice that had received the TR-R-RNA developed RRV-binding antibodies (Figure 4C). Immunization with the combination of TR-C- and TR-R-RNA induced the development of CHIKV- and RRV-binding antibodies. However, the mean total antibody titers were reduced in comparison to the respective single vaccinations. The antibody titers after vaccination with a single TR-RNA were comparable in both doses studied. In contrast, after the bivalent vaccine, a higher endpoint titer and boost following the second vaccination was only noted at the higher RNA dose.

Interestingly, some TR-C-RNA-vaccinated mice had RRV-binding antibodies and some TR-R-RNA-vaccinated mice had CHIKV-binding antibodies, although the induction was not significant. To further evaluate the potential maturation of pan-alphavirus-binding antibodies, MAYV-binding antibodies were also determined. Some mice had MAYV-binding IgG; however, compared to control mice, the observed antibody titers were not significant (Figure 4C).

**Figure 5:**
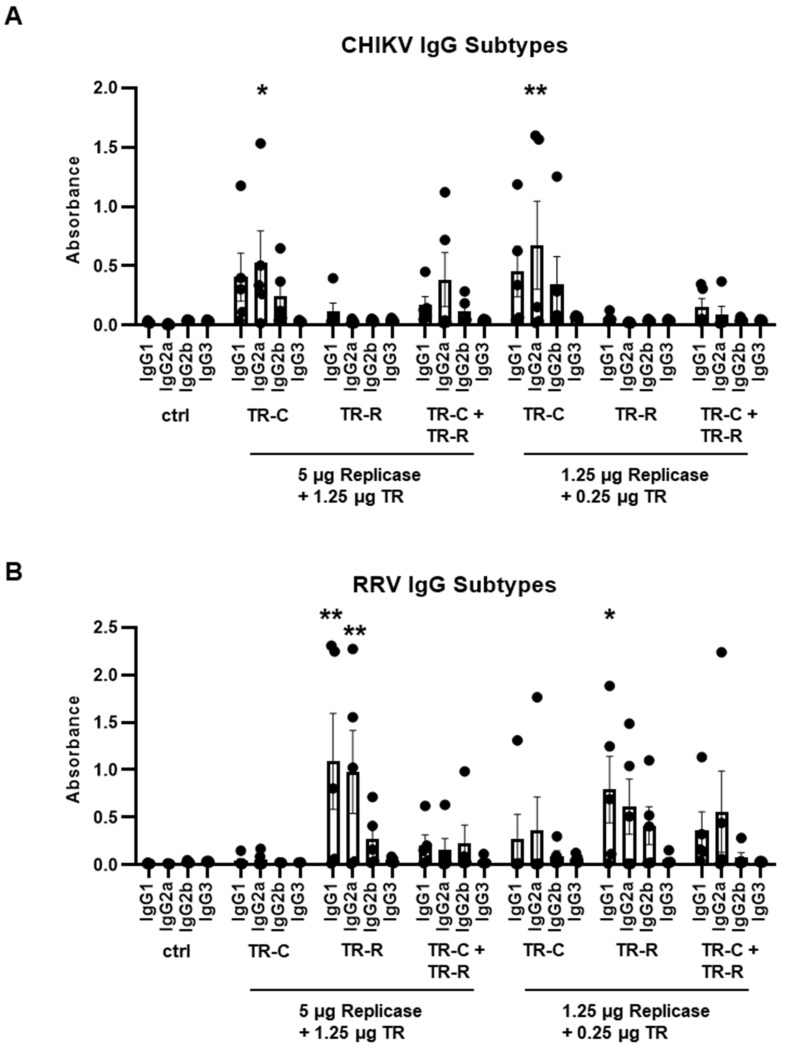
IgG subtypes after taRNA vaccination. (**A**): CHIKV-specific IgG subtypes and (**B**): RRV-specific IgG subtypes on day 56 as determined by ELISA. The mean values ± SEM of each group (*n* = 5) are depicted. For statistical analysis, a two-way ANOVA with Dunnett’s multiple comparison post-test was performed (* *p* < 0.05; ** *p* < 0.01).

To further characterize the induced humoral immune responses, the CHIKV- and RRV-specific IgG subtypes on day 56 were determined. IgG2a and IgG2b can protect against alphavirus infections via Fc receptor functions [32]. Indeed, after vaccination with replicase-RNA and TR-C-RNA, a higher frequency of IgG2a over IgG1 antibodies was observed (Figure 5A). Similarly, vaccination with 5 µg replicase-RNA and 1.25 µg TR-C- and TR-R-RNA induced the development of CHIKV-specific IgG2a antibodies, although with a lower titer. Vaccination with replicase-RNA and TR-R-RNA resulted in similar levels of IgG1 and IgG2a antibodies (Figure 5B). A comparable antibody profile was again observed for the bivalent vaccination with a lower overall response rate. No CHIKV- or RRV-specific IgG3 responses were detected after any vaccination.

**Figure 6:**
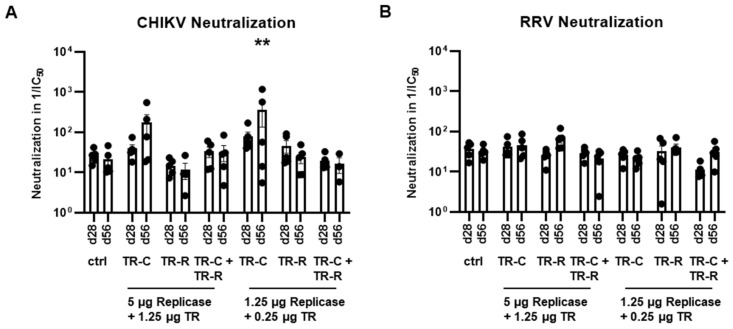
Neutralization capacity after taRNA vaccination. (**A**): Neutralization of CHIKV- and (**B**): RRV-pseudotyped lentiviral vector particles by mouse sera. The neutralization capacity is shown as reciprocal IC_50_ values. The mean values ± SEM of each group (*n* = 5) are depicted. For statistical analysis, a two-way ANOVA with Dunnett’s multiple comparison post-test was performed (** *p* < 0.01).

Next, neutralizing antibodies were assessed using pseudotyped lentiviral vector particles. At day 56 after vaccination with replicase-RNA and TR-C-RNA, CHIKV-neutralizing antibodies were detected for both tested vaccine doses (Figure 6A). If mice had received TR-C- and TR-R-RNA, no CHIKV neutralization could be observed. Moreover, no RRV neutralization was found after either single or bivalent taRNA vaccination (Figure 6B).

### 3.4. T Cell Responses after taRNA Vaccination

**Figure 7:**
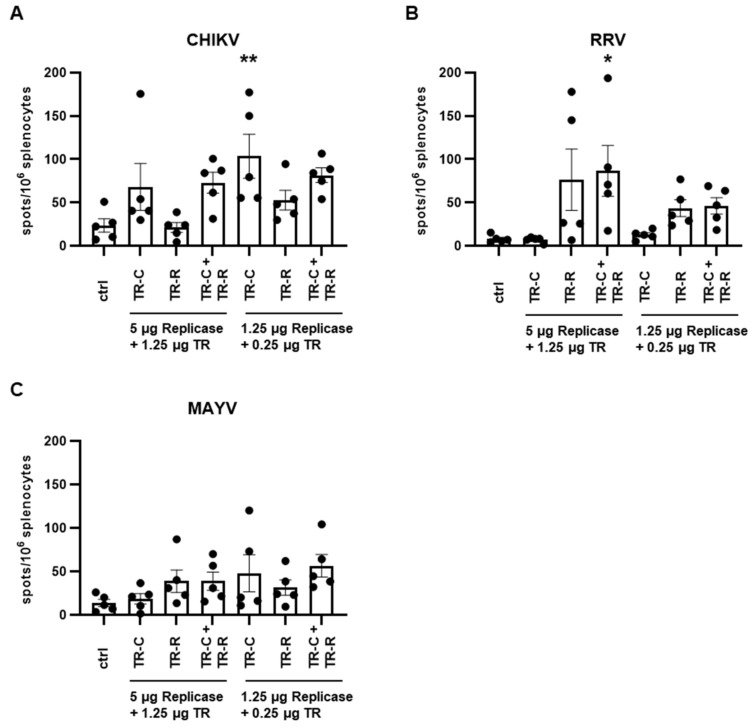
T cell responses after taRNA vaccination. Mouse splenocytes were isolated on day 56 and re-stimulated with (**A**): CHIKV, (**B**): RRV, and (**C**): MAYV and IFN-γ-secreting T cells were then quantified by ELISpot. The mean values ± SEM of each group (*n* = 5) are depicted. For statistical analysis, a one-way ANOVA with Dunnett’s multiple comparison post-test was performed (* *p* < 0.05; ** *p* < 0.01).

To further analyze the immune responses after taRNA vaccination, splenocytes were harvested on day 56 and re-stimulated with CHIKV, RRV, or MAYV. IFN-γ-secreting T cell were then quantified by ELISpot. Immunization with the TR-C-RNA induced significant amounts of CHIKV-reactive T cells (Figure 7A) and immunization with the TR-R-RNA induced RRV-reactive T cells (Figure 7B). After vaccination with both TR-RNAs, CHIKV- and RRV-reactive T cells were induced. Importantly, the T cell titers after the bivalent vaccination were comparable to those observed after vaccination with a single TR-RNA. The RNA dose did not significantly influence the T cell titers. To analyze potential pan-alphavirus T cell responses, the splenocytes were re-stimulated with the alphavirus MAYV. A low frequency of MAYV-reactive T cells was observed in the vaccinated groups, although the increases in titer compared to those of control mice were not statistically significant (Figure 7C).

## 4. Discussion

Alphaviruses such as the human pathogenic CHIKV, RRV, and MAYV can cause massive outbreaks of acute infections with high fever, myalgia, and polyarthralgia. The arthritogenic symptoms of patients can become persistent and last for months to years. However, no alphavirus vaccine has so far been licensed. Here, we show that a bivalent taRNA vaccine candidate against CHIKV and RRV was able to induce humoral and cellular immune responses in mice.

In vitro, the TR-RNAs were amplified by the CHIKV replicase. Importantly, after transfection of both TR-RNAs, each RNA was efficiently amplified with only a slight preference for the TR-C-RNA. In contrast, a clear preference for a shorter TR-RNA has been observed in our previous studies [15]. Here, we designed TR-RNAs with similar lengths and indeed, we could observe amplification of both TR-RNAs. Thus, to extend the taRNA vaccine system with further TR-RNAs to generate multivalent vaccines, RNAs should preferably be of comparable length.

The TR-RNA was efficiently amplified and the antigenic CHIKV E2 and RRV E2 proteins were expressed at similar levels to those in CHIKV/RRV-infected cells. Furthermore, the CHIKV and RRV E2 proteins were released into the cell supernatant without the expression of an alphavirus capsid protein as we had observed before [15]. This is in line with previous studies demonstrating that capsid-deleted alphaviruses can assemble into microparticles [33,34]. These have a similar antigenicity to the respective wild-type virus and thus might potently stimulate humoral immune responses.

Indeed, intradermal taRNA vaccination could induce humoral and cellular immune responses in mice. The antibody titers and neutralization activity after the CHIKV only vaccination were comparable to the observed immune responses of our previous study [15]. The bivalent taRNA vaccination was able to generate CHIKV- and RRV-specific antibodies. However, compared to the respective single vaccinations, the endpoint titers were reduced. In addition to the total antibody titers, the bivalent vaccination also showed reduced neutralizing antibody responses. The total TR-RNA amount was kept stable in the experiment, resulting in half the dose of each TR-RNA in the bivalent vaccine candidate. The lower amount of TR-RNA might have been responsible for the reduced humoral immune response. On the other hand, immune interferences have previously been shown to affect antibody responses after alphavirus vaccinations. One study in humans demonstrated that, after a first vaccination against VEEV and a subsequent vaccination against CHIKV, only 46% responded with CHIKV-neutralizing antibodies [35]. Moreover, vaccination against EEEV and WEEV on the same day reduced the response rates [24]. However, a trivalent virus-like particle vaccine against WEEV, EEEV, and VEEV was able to induce immune responses against all three viruses in the majority of participants in a phase-I clinical trial [22].

In addition to neutralizing antibodies, non-neutralizing antibodies of the IgG2a/c subtype are able to protect mice against MAYV infection [31]. Importantly, we observed that the pattern of the IgG response here was not altered by the bivalent vaccination in comparison to the single vaccinations. Although the overall titers were reduced, IgG2a and IgG2b antibodies were detected, which might mediate protection against CHIKV or RRV infection in vivo. Unfortunately, no clear correlates of protection against alphavirus infections have so far been defined [36]. Neutralizing antibodies usually correlate with protection against infection or clearance of the virus; however, non-neutralizing antibodies and T cell responses can play a part in the protection against virus infections. CD8+, NKT, and gamma-delta (γδ) T cells can contribute to protection and disease progression, even if they do not appear to play an important role after natural alphavirus infection [37]. Here, we observed that, in contrast to the antibody responses, the bivalent taRNA vaccination induced potent T cell responses without immune interference effects. Further studies are needed to evaluate the impact of TR-RNA amounts or immune interference mechanisms on humoral and cellular immune responses.

Suboptimal neutralizing or non-neutralizing cross-reactive antibodies can lead to enhanced disease after dengue virus infections due to antibody-dependent enhancement (ADE), which is a major challenge for vaccine development [38]. In contrast, alphavirus antibody responses have been described not to promote ADE, but instead to be cross-protective. For example, mice which had been infected with CHIKV were partially protected against subsequent MAYV infections [39,40]. Moreover, a CHIKV vaccine candidate was able to protect mice against o’nyong-nyong virus (ONNV) infections [41]. Here, we evaluated potential cross-reactivity to other alphaviruses by analyzing the immune responses against MAYV. The amino acid identity between the studied viruses is similar with 60.3% between CHIKV and MAYV, 61.8% between CHIKV and RRV, and 65.9% between RRV and MAYV [42,43]. Indeed, low levels of cross-reactive antibodies and T cells to MAYV were found; however, the responses were not statistically significant. CHIKV vaccination might induce more potent ONNV cross-reactive immune responses due to the close relation (86.3% identity). Still, monoclonal antibodies, which are cross-reactive to all three tested alphaviruses, have been described before [42]. For efficient cross-protection against heterologous alphaviruses, much higher immune responses induced by an elevated vaccine dose or multiple vaccinations might be required [44].

Although very promising, the taRNA vaccine platform should be further optimized. In this study, we used unmodified RNA, but the replicase-RNA could be nucleoside modified. Nucleoside modification reduces the innate immune responses and thus leads to a higher antigen expression and superior immune responses [45]. We applied the taRNA vaccine intradermally without sophisticated formulation, which is inherently inefficient. Formulation with, e.g., lipid nanoparticles could improve RNA stability and uptake into cells. A higher in vivo antigen expression obtained by different optimization strategies might also increase the induced immune responses and enable more potent neutralizing antibody responses.

In our previous study, the single taRNA CHIKV vaccine candidate protected mice against a high dose CHIKV challenge [15]. Still, protection by the bivalent taRNA vaccine candidate needs to be further evaluated by RRV or CHIKV challenge infections. Additionally, potential cross-protection against ONNV or MAYV could be studied. Alternatively, further TR-RNAs could be added for the development of a multivalent alphavirus vaccine candidate that may be able to protect against infection with Old and New World alphaviruses.

## 5. Conclusions

In summary, taRNA vaccines are a promising vaccination platform, which can be used to design multivalent alphavirus vaccine candidates. However, further optimizations are required for clinical application and the effects of potential immune interference mechanisms on antibody responses need to be monitored.

## Data Availability

The data presented in this study are available in the article.

## Supplementary Materials

The following supporting information can be downloaded at: https://www.mdpi.com/article/10.3390/vaccines10091374/s1, Supplementary Figure S1: Sequence of pTR-R. Supplementary Figures S2–S6: Uncropped blots and densitometry readings of blots shown in Figure 3A,B.
