# Supplementary material for: A Bivalent Trans-Amplifying RNA Vaccine Candidate Induces Potent Chikungunya and Ross River Virus Specific Immune Responses
Source: Vaccines (Basel). 2022 Aug 23;10(9):1374. doi: 10.3390/vaccines10091374 (PMC9503900; doi:10.3390/vaccines10091374)
Supplement: Supplementary file 1 [file vaccines-10-01374-s001.zip › vaccines-1859285-supplementary.pdf]

Supplementary Material

A Bivalent Trans-Amplifying RNA Vaccine Candidate Induces Potent Chikungunya and Ross River Virus Specific Immune Responses

Christin Schmidt [1], Florian D. Hastert [1], Julia Gerbeth [1], Tim Beissert [2], Ugur Sahin [2,3], Mario Perkovic [2,*] and Barbara S. Schnierle [1,*]

[1] Department of Virology, Paul-Ehrlich-Institut, Paul-Ehrlich Strasse 51-59, 63225 Langen, Germany;
christin.schmidt@pei.de (C.S.); florian.hastert@pei.de (F.H.); julia.gerbeth@pei.de (J.G.)

[2] TRON (Translational Oncology at the University Medical Center), Johannes Gutenberg University Mainz, Freiligrathstraße 12, 55131 Mainz, Germany; tim.beissert@tron-mainz.de (T.B.);
ugur.sahin@tron-mainz.de (U.S.)

[3] Research Center for Immunotherapy (FZI), University Medical Center at the Johannes Gutenberg University, Langenbeckstr. 1, 55131 Mainz, Germany

* Correspondence: mario.perkovic@tron-mainz.de (M.P.); barbara.schnierle@pei.de (B.S.S.)

TR-R

ATGGCTGCGTGAGACACACGTAGCCTACCAGTTTCTTACTGCTCTACTCTGCAAAGCAAG

AGATTAATAACCCATCATGGATCCTGTGTACGTGGACATAGACGCTGACAGCGCCTTTTT

GAAGGCCCTGCAACGTGCGTACCCCATGTTTGAGGTGGAACCAAGGCAGGTCACACCGAA

TGACCATGCTAATGCTAGAGCGTTCTCGCATCTAGCTATAAAACTAATAGAGCAGGAAAT

TGACCCCGACTCAACCATCCTGGATATCGGCAGTGCGCCAGCAAGGAGGATGATGTCGGA

CAGGAAGTAACACCGGTCCAGGTCATTTACAACAGAAGTCAGTACGCCAGTCAGTGCTGC

CGGTGAACACCCTGGAGGAAGTCCACGAGGAGAAGTGTTACCCACCTAAGCTGGATGAAG

CAAAGGAGCAACTATTACTTAAGAAACTCCAGGAGAGTGCATCCATGGCCAACAGAAGCA

GGTATCAGTCGCGCAAAGTAGAAAACATGAAAGCAGCAATCATCCAGAGACTAAAGAGAG

GCTGTAGACTATACTTAATGTCAGAGACCCCAAAAGTCCCTACTTACCGGACTACATATC

CGGCGCCTGTGTACTCGCCTCCGATCAACGTCCGATTGTCCAATCCCGAGTCCGCAGTGG

CAGCATGCAATGAGTTCTTAGCTAGAAACTATCCAACTGTCTCATCATACCAAATTACCG

ACGAGTATGATGCATATCTAGACATGGTGGACGGGTCGGAGAGTTGCCTGGACCGAGCGA

CATTCAATCCGTCAAAACTCAGGAGCTACCCGAAACAGCACGCTTACCACGCGCCCTCCA

TCAGAAGCGCTGTACCGTCCCCATTCCAGAACACACTACAGAATGTACTGGCAGCAGCCA

CGAAAAGAAACTGCAACGTCACACAGATGAGGGAATTACCCACTTTGGACTCAGCAGTAT

TCAACGTGGAGTGTTTCAAAAAATTCGCATGCAACCAAGAATACTGGGAAGAATTTGCTG

CCAGCCCTATTAGGATAACAACTGAGAATTTAGCAACCTATGTTACTAAACTAAAAGGGC

CAAAAGCAGCAGCGCTATTCGCAAAAACCCATAATCTACTGCCACTACAGGAAGTACCAA

TGGATAGGTTCACAGTAGATATGAAAAGGGACGTAAAGGTGACTCCTGGTACAAAGCATA

CAGAGGAAAGACCTAAGGTGCAGGTTATACAGGCGGCTGAACCCTTGGCGACAGCATACC

TATGTGGGATTCACAGAGAGCTGGTTAGGAGGCTGAACGCCGTCCTCCTACCCAATGTAC

ATACACTATTTGACATGTCTGCCGAGGATTTCGATGCCATCATAGCCGCACACTTTAAGC

CAGGAGACACTGTTTTGGAAACGGACATAGCCTCCTTTGATAAGAGCCAAGATGATTCAC

TTGCGCTTACTGCTTTGATGCTGTTAGAGGATTTAGGGGTGGATCACTCCCTGCTGGACT

TGATAGAGGCTGCTTTCGGAGAGATTTCCAGCTGTCACCTACCGACAGGTACGCGCTTCA

AGTTCGGCGCCATGATGAAATCAGGTATGTTCCTAACTCTGTTCGTCAACACATTGTTAA

ACATCACCATCGCCAGCCGAGTGCTGGAAGATCGTCTGACAAAATCCGCGTGCGCGGCCT

TCATCGGCGACGACAACATAATACATGGAGTCGTCTCCGATGAATTGATGGCAGCCAGAT
GTGCCACTTGGATGAACATGGAAGTGAAGATCATAGATGCAGTTGTATCCTTGAAAGCCC
CTTACTTTTGTGGAGGGTTTATACTGCACGATACTGTGACAGGAACAGCTTGCAGAGTGG
CAGACCCGCTAAAAAGGCTTTTTAAACTGGGCAAACCGCTAGCGGCAGGTGACGAACAAG
ATGAAGATAGAAGACGAGCGCTGGCTGACGAAGTGATCAGATGGCAACGAACAGGGCTAA
TTGATGAGCTGGAGAAAGCGGTATACTCTAGGTACGAAGTGCAGGGTATATCAGTTGTGG
TAATGTCCATGGCCACCTTTGCAAGCTCCAGATCCAACTTCGAGAAGCTCAGAGGACCCG
TCATAACTTTGTACGGCGGTCCTAAATAGGTACGCACTACAGCTACCTATTTTGCAGAAG
CCGACAGCAAGTATCTAAACACTAATCAGCTACACTAGTGCCACCATGTCTGCCGCGCTG
ATGATGTGTATCCTTGCCAACACCTCTTTCCCCTGCTCATCACCTCCCTGCTACCCCTGC
TGCTACGAAAAACAGCCAGAACAGACACTGCGGATGCTGGAAGACAATGTGAATAGACCA
GGGTACTATGAGCTACTGGAAGCGTCCATGACATGCAGAAACAGATCACGCCACCGCCGT
AGTGTAACAGAGCACTTCAATGTGTATAAGGCTACTAGACCGTACTTAGCGTATTGCGCT
GACTGTGGGGACGGGTACTTCTGCTATAGCCCAGTTGCTATCGAGAAGATCCGAGATGAG
GCGTCTGACGGCATGCTCAAGATCCAAGTCTCCGCCCAAATAGGTCTGGACAAGGCAGGT
ACCCACGCCCACACGAAGATCCGATATATGGCTGGTCATGATGTTCAGGAATCTAAGAGA
GATTCCTTGAGGGTGTACACGTCCGCAGCGTGCTCTATACATGGGACGATGGGACACTTC
ATCGTCGCACATTGTCCGCCAGGCGACTACCTCAAGGTTTCGTTCGAGGACGCAGACTCA
CACGTGAAGGCATGTAAGGTCCAATACAAGCACGACCCATTGCCGGTGGGTAGAGAGAAG
TTCGTGGTTAGACCCCACTTTGGCGTAGAGCTGCCATGCACCTCATACCAGCTGACAACA
GCTCCCACCGACGAGGAGATCGACATGCACACACCGCCAGATATACCGGATCGCACCCTG
CTATCACAGACGGCGGGCAACGTCAAAATAACAGCAGGCGGCAGGACTATCAGGTACAAT
TGTACCTGTGGCCGTGACAACGTAGGCACTACCAGTACTGACAAGACCATCAACACATGC
AAGATTGACCAATGCCATGCTGCCGTTACCAGCCATGACAAATGGCAATTTACCTCTCCA
TTTGTTCCCAGGGCTGATCAGACAGCTAGGAGGGGCAAAGTGCATGTTCCATTCCCTTTG
ACTAACGTCACCTGCCGAGTGCCGTTGGCTCGAGCGCCGGATGTCACCTATGGTAAGAAG
GAGGTGACCCTGAGATTACACCCAGATCATCCGACGCTGTTCTCCTATAGGAGTTTAGGA
GCCGAACCGCACCCGTACGAGGAGTGGGTTGACAAGTTCTCTGAGCGCATCATCCCAGTG
ACGGAAGAAGGGATTGAGTACCAGTGGGGCAACAACCCGCCGGTCCGCCTATGGGCGCAA

CTGACGACCGAGGGCAAACCCCATGGCTGGCCACATGAAATCATTCAGTACTATTATGGA

CTATACCCCGCCGCCACCATTGCCGCAGTATCCGGGGCGAGTCTGATGGCCCTCCTAACT

CTAGCGGCCACATGCTGCATGCTGGCCACCGCGAGGAGAAAGTGCCTAACACCATACGCC

TTGACGCCAGGAGCGGTGGTACCGTTGACACTGGGGCTGCTTTGCTGCGCACCGAGGGCG

AACGCAGCATCATTCGCTGAGACTATGGCATATCTGTGGGACGAGAACAAAACCCTCTTT

TGGATGGAATTCGCGGCCCCAGCCGCAGCGCTTGCTTTGCTGGCATGCTGTATCAAAAGC

CTGATCTGCTGTTGTAAGCCATTTTCTTTTTTAGTGTTACTGAGCCTGGGAGCCTCCGCA

AAAGCTTACGAGCACACAGCCACAATTCCGAATGTGGTGGGGTTCCCGTATAAGGCTCAC

ATTGAAAGGAATGGCTTCTCGCCCATGACTCTGCAGCTTGAAGTGGTGGAGACAAGCTTG

GAACCCACACTTAACCTGGAGTACATTACCTGCGAATACAAGACGGTGGTCCCTTCGCCA

TTCATCAAATGTTGCGGAACATCAGAATGCTCATCCAAGGAGCAGCCAGACTACCAATGC

AAGGTGTACACGGGTGTATACCCATTCATGTGGGGTGGAGCCTACTGTTTCTGCGACTCC

GAGAACACGCAGCTCAGCGAGGCCTATGTCGACAGGTCAGACGTTTGCAAACATGATCAC

GCATCGGCCTACAAGGCACACACGGCCTCTCTAAAAGCAACAATCAGGATCAGTTATGGC

ACCATCAACCAGACCACCGAGGCCTTCGTTAATGGTGAACACGCGGTCAACGTGGGCGGA

AGCAAGTTCATCTTTGGACCGATCTCAACAGCTTGGTCACCGTTCGACAATAAAATTGTC

GTGTATAAAGATGATGTCTACAACCAGGACTTCCCACCCTACGGATCAGGCCAGCCGGGT

AGATTCGGAGACATTCAGAGCAGGACAGTGGAGAGCAAAGACTTGTATGCCAACACGGCC

CTAAAACTCTCAAGACCATCACCCGGGGTTGTGCATGTGCCATACACGCAAACACCATCC

GGATTTAAATATTGGCTGAAGGAGAAAGGATCTTCATTGAATACAAAGGCCCCTTTTGGC

TGCAAGATAAAGACCAATCCAGTCAGAGCCATGGATTGTGCAGTTGGCAGTATACCTGTG

TCGATGGACATACCTGACAGTGCATTCACACGAGTGGTAGATGCCCCGGCTGTAACAGAC

CTGAGCTGCCAGGTAGTGGTCTGTACACACTCCTCCGATTTCGGAGGAGTTGCCACATTG

TCTTACAAAACGGACAAACCCGGCAAGTGCGCTGTCCACTCACATTCCAACGTCGCAACG

TTGCAAGAGGCGACGGTGGATGTCAAGGAGGATGGCAAGGTCACAGTGCACTTTTCCACG

GCGTCCGCCTCCCCGGCCTTCAAAGTGTCCGTCTGTGACGCAAAAACAACGTGCACGGCG

GCGTGCGAGCCTCCAAAAGACCACATCGTCCCTTATGGGGCGAGCCATAACAACCAGGTC

TTTCCGGACATGTCAGGAACTGCGATGACGTGGGTGCAGAGGCTGGCCAGTGGGTTAGGT

GGGCTGGCTCTCATCGCGGTGGTTGTGCTGGTCTTGGTAACCTGCATAACAATGCGTCGG

TAACCTCGAGGCGGCCGCTTGACAATTAAGTATGAAGGTATATGTGTCCCCTAAGAGACA

CACTGTACATAGCAAATAATCTATAGATCAAAGGGCTACGCAACCCCTGAATAGTAACAA

AATACAAAATCACTAAAAATTATAAAAACAGAAAAATACATAAATAGGTATACGTGTCCC

CTAAGAGACACATTGTATGTAGGTGATAAGTATAGATCAAAGGGCCGAATAACCCCTGAA

TAGTAACAAAATATGAAAATCAATAAAAATCATAAAATAGAAAAACCATAAACAGAAGTA

GTTCAAAGGGCTATAAAACCCCTGAATAGTAACAAAACATAAAATTAATAAAAATCAAAT

GAATACCATAATTGGCAAACGGAAGAGATGTAGGTACTTAAGCTTCCTAAAAGCAGCCGA

ACTCACTTTGAGAAGTAGGCATAGCATACCGAACTCTTCCACGATTCTCCGAACCCACAG

GGACGTAGGAGATGTTATTTTGTTTTTAATATTTC

Figure S1. Sequence of pTR-R.

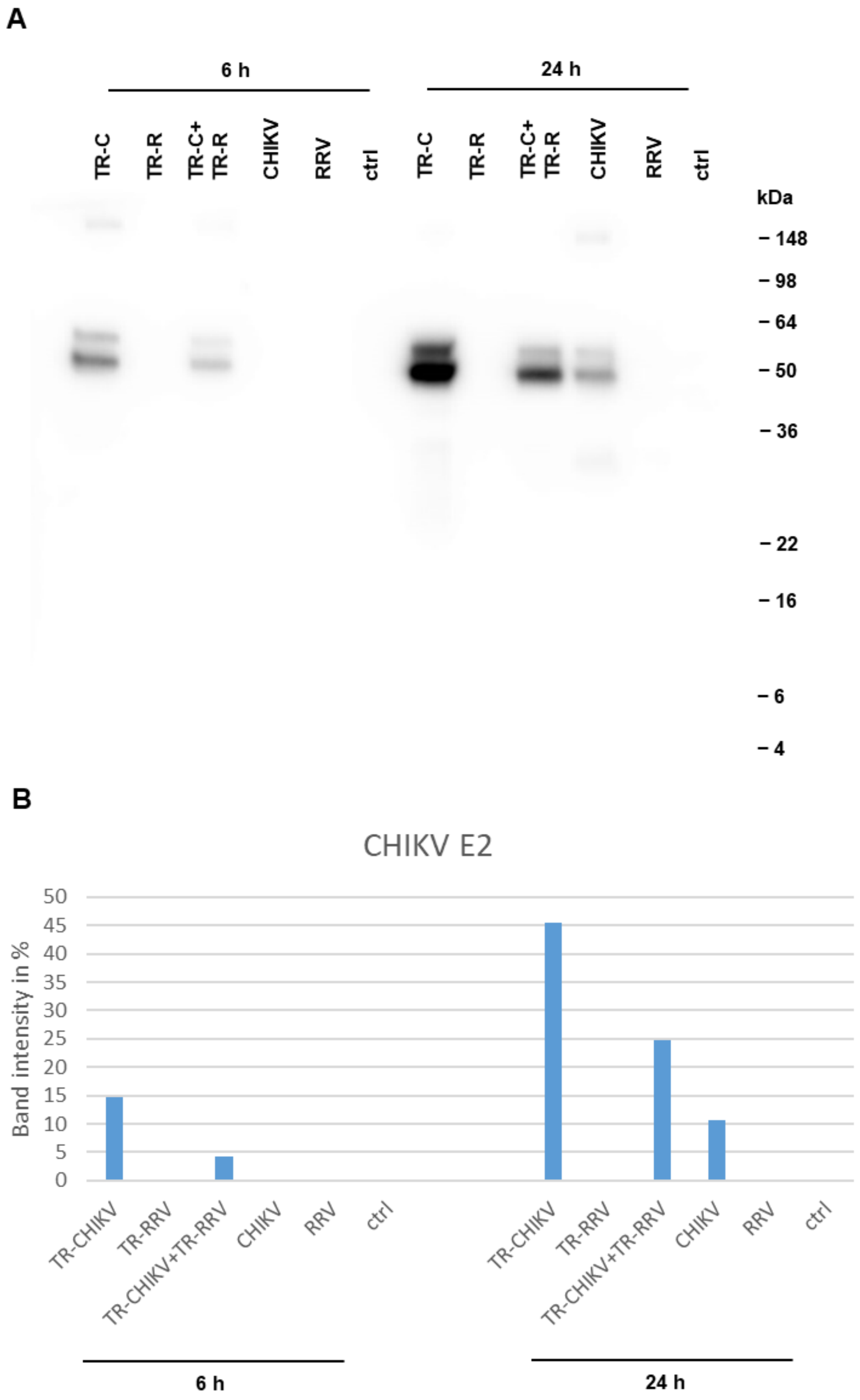

Figure S2. (A) Uncropped western blot of Figure 3A showing CHIKV E2 protein expression in cellular lysates 6 h and 24 h after transfection. (B) Band intensity of CHIKV E2 protein expression given in relative percentage.

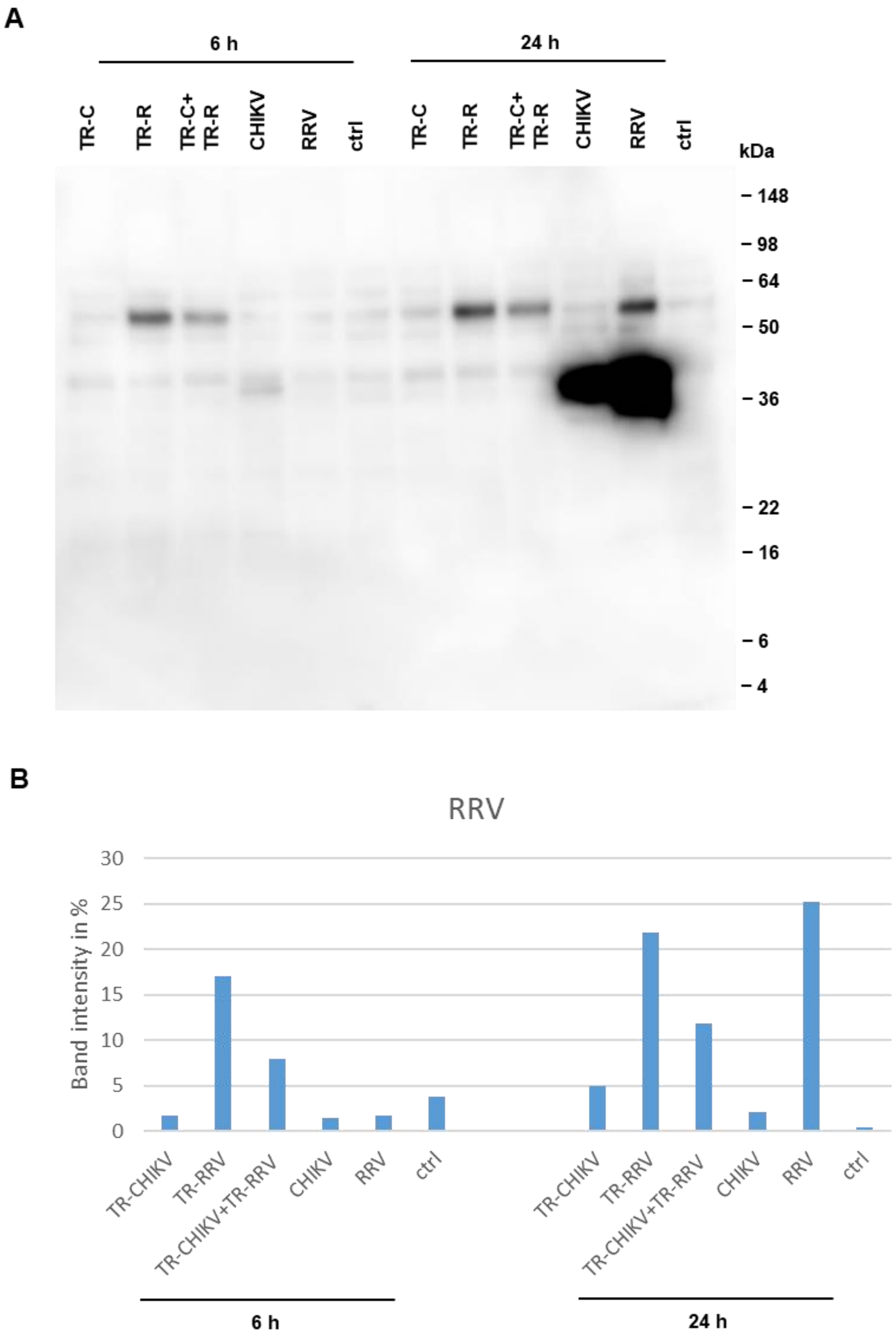

Figure S3. (A) Uncropped western blot of Figure 3A showing RRV protein expression in cellular lysates 6 h and 24 h after transfection. (B) Band intensity of RRV protein expression given in relative percentage.

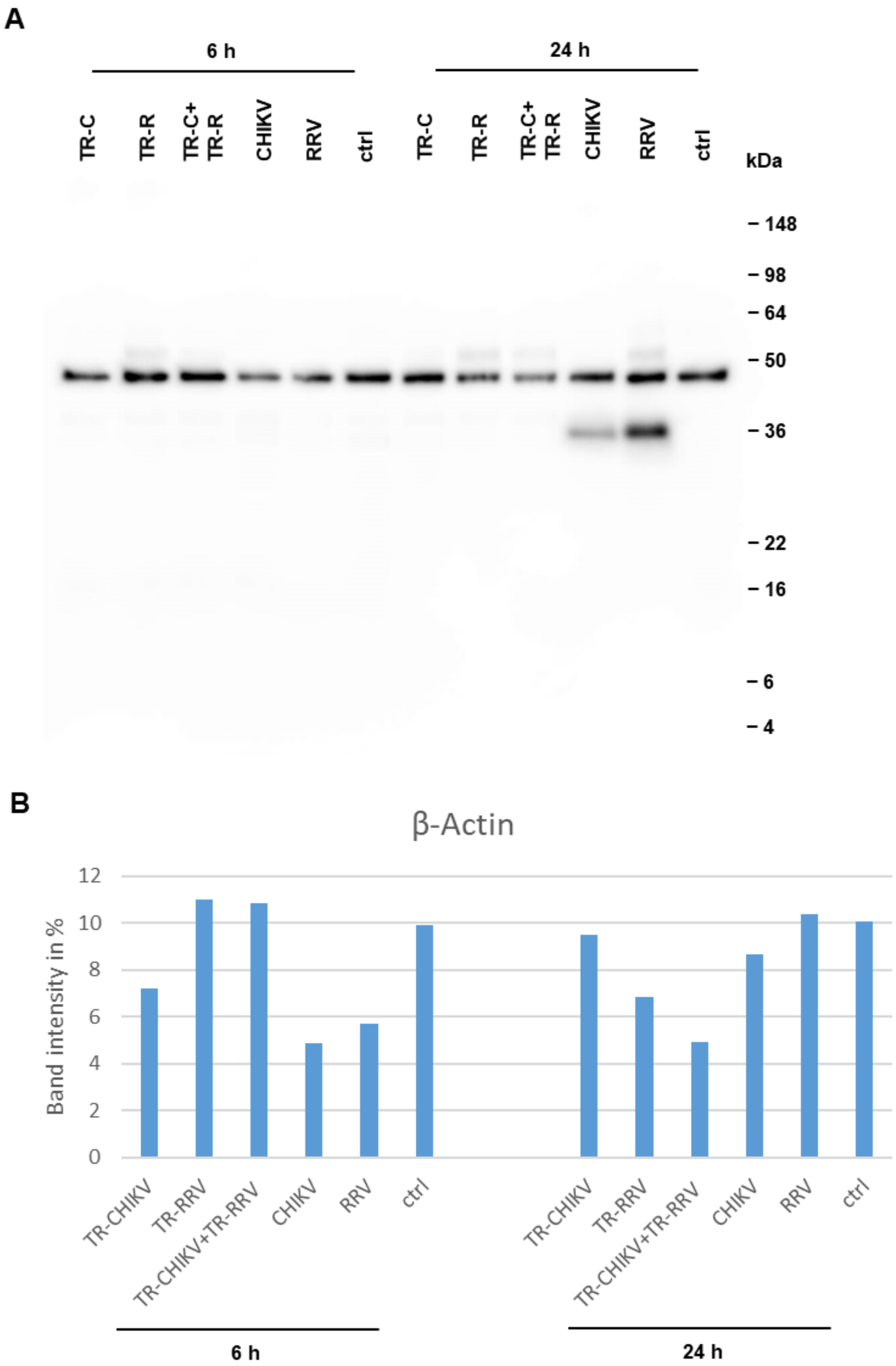

Figure S4. (A) Uncropped western blot of Figure 3A showing β-actin protein expression in cellular lysates 6 h and 24 h after transfection. (B) Band intensity of β-actin protein expression given in relative percentage.

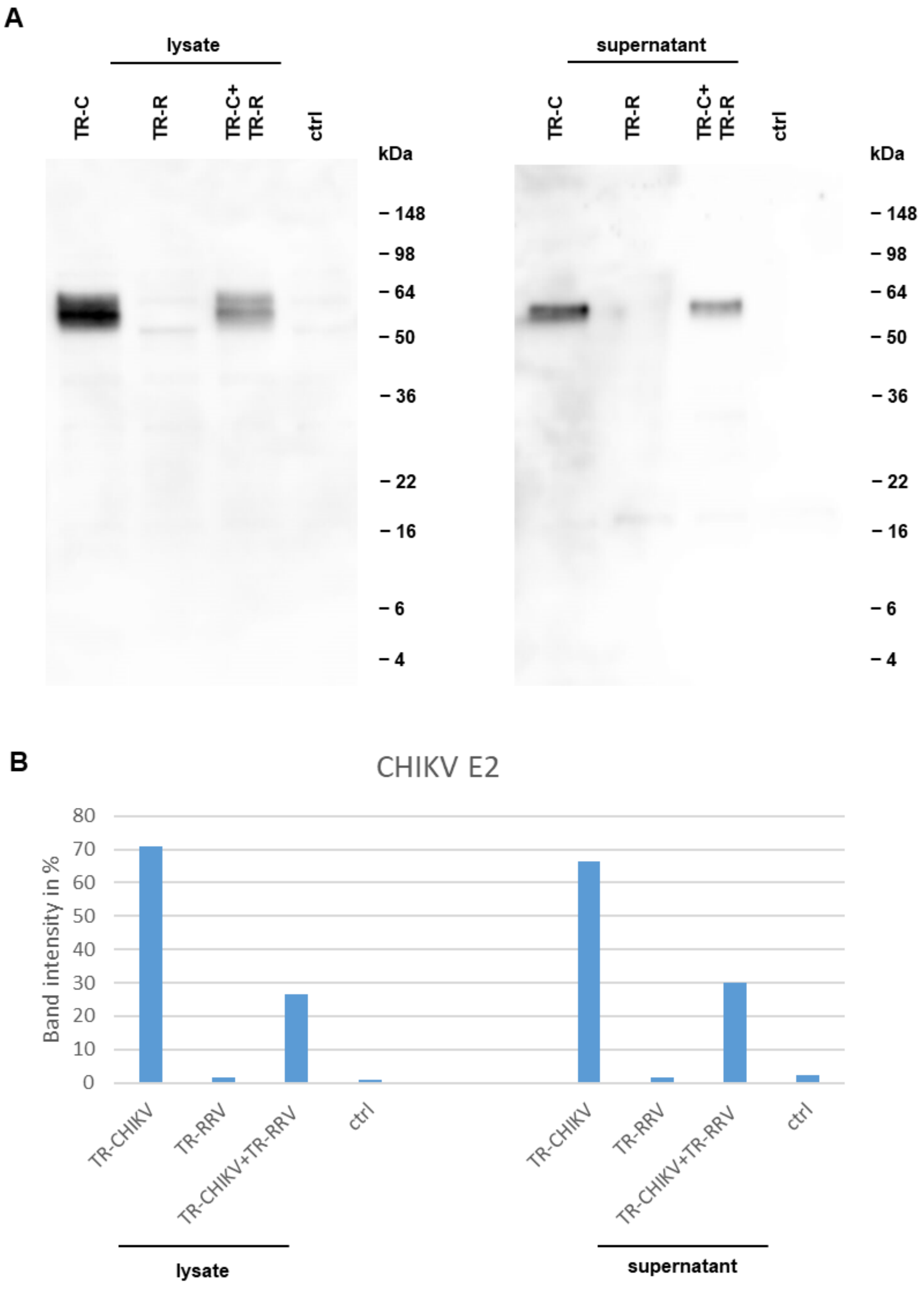

Figure S5. (A) Uncropped western blot of Figure 3B showing CHIKV E2 protein expression in cellular lysates and concentrated supernatants 48 h after transfection. (B) Band intensity of CHIKV E2 protein expression given in relative percentage.

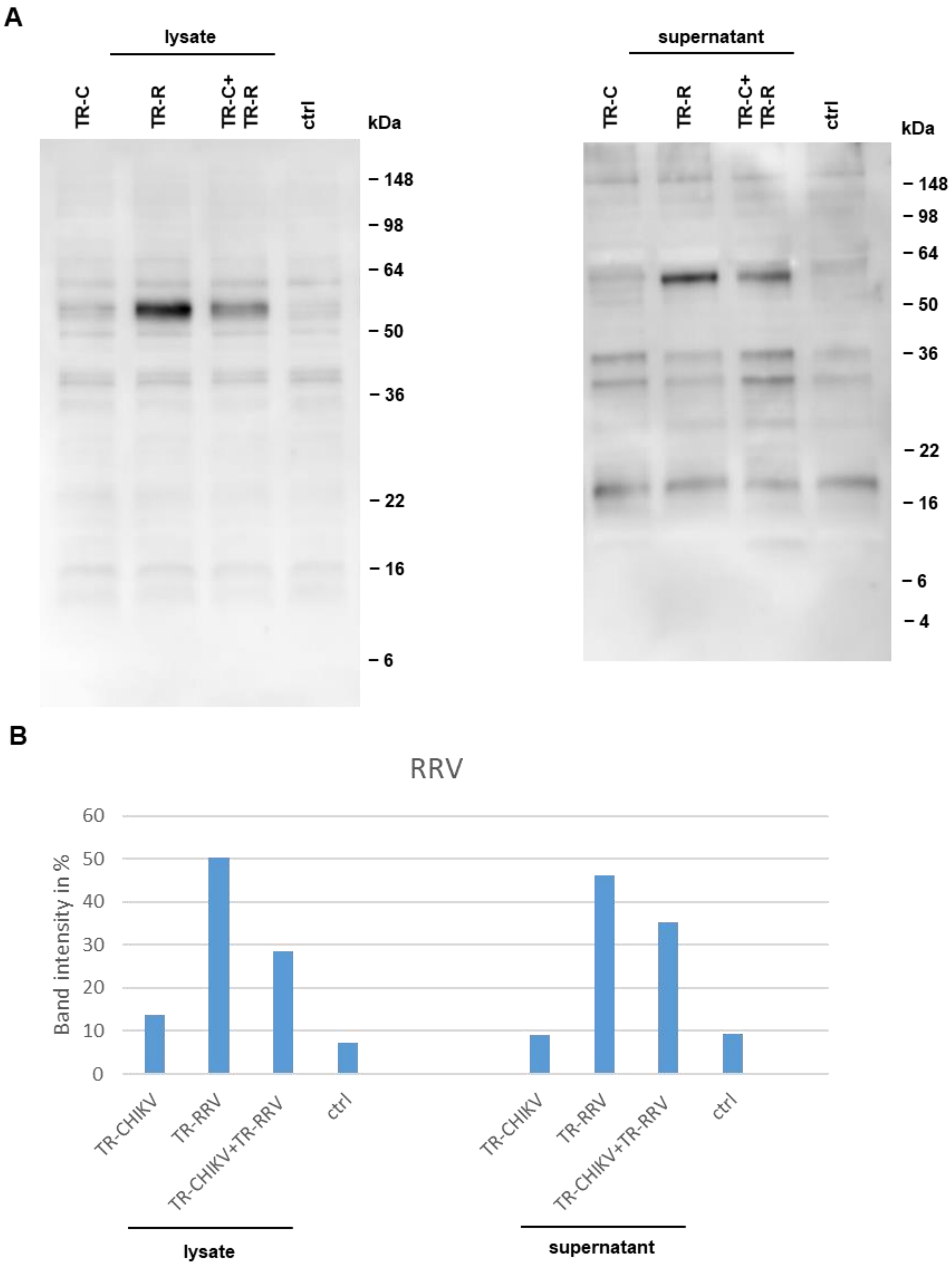

Figure S6. (A) Uncropped western blot of Figure 3B showing RRV protein expression in cellular lysates and concentrated supernatants 48 h after transfection. (B) Band intensity of RRV protein expression given in relative percentage.

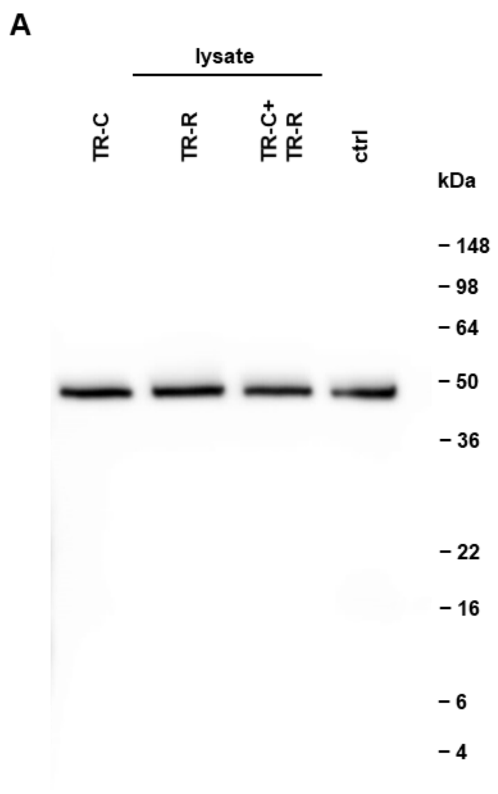

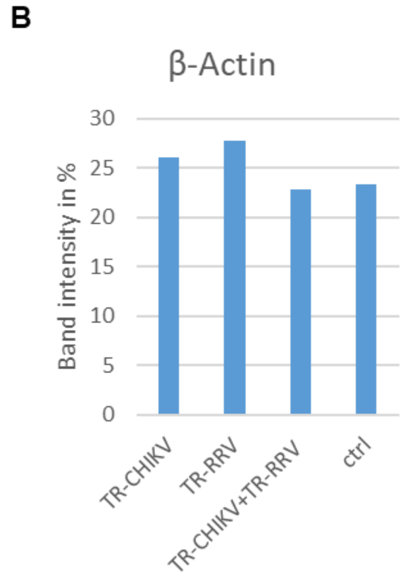

Figure S7. (A) Uncropped western blot of Figure 3B showing β-actin protein expression in cellular lysates 48 h after transfection. (B) Band intensity of β-actin protein expression given in relative percentage.
